# Additive Nano-Lithography with Focused Soft X-rays: Basics, Challenges, and Opportunities

**DOI:** 10.3390/mi10120834

**Published:** 2019-11-30

**Authors:** Andreas Späth

**Affiliations:** Friedrich-Alexander-University Erlangen-Nuremberg, Physical Chemistry II, Egerlandstraße 3, 91058 Erlangen, Germany; andreas.spaeth@fau.de

**Keywords:** direct-write nanofabrication, additive nanofabrication, X-ray lithography, X-ray microscopy

## Abstract

Focused soft X-ray beam induced deposition (FXBID) is a novel technique for direct-write nanofabrication of metallic nanostructures from metal organic precursor gases. It combines the established concepts of focused electron beam induced processing (FEBIP) and X-ray lithography (XRL). The present setup is based on a scanning transmission X-ray microscope (STXM) equipped with a gas flow cell to provide metal organic precursor molecules towards the intended deposition zone. Fundamentals of X-ray microscopy instrumentation and X-ray radiation chemistry relevant for FXBID development are presented in a comprehensive form. Recently published proof-of-concept studies on initial experiments on FXBID nanolithography are reviewed for an overview on current progress and proposed advances of nanofabrication performance. Potential applications and advantages of FXBID are discussed with respect to competing electron/ion based techniques.

## 1. Introduction

Several recent studies have demonstrated that focused electron beam induced processing (FEBIP) has high potential for the controlled bottom-up fabrication of metallic 3D nanostructures [1,2,3,4,5,6,7,8,9,10,11,12,13,14]. Furthermore, the basic processes during FEBIP are understood to more and more detail leading to developments towards more diverse, cleaner and more defined deposits for various potential applications [7,15,16,17,18]. The method employs a focused electron beam to induce local dissociation of surface-absorbed metal organic precursor molecules supplied from the gas-phase. While the dissociation products containing the metal center of the initial complex are non-volatile and will stay on the substrate, volatile portions from the ligands are pumped off by the vacuum system. Dissociation is generated mainly by low- and medium-energy secondary or backscattered electrons which have a high interaction cross-section with matter. Depending on the substrate and the chemical composition of the targeted nanostructure, several in-situ and ex-situ cleaning procedures have been developed [14,19,20]. 

The concept of focused soft X-ray beam induced deposition (FXBID) is basically a combination of FEBIP and X-ray lithography (XRL) [21,22]. FEBIP and XRL are based on very similar fundamental principles. The radiation chemistry in XRL is also mainly caused by low-energy secondary electrons evolving from the decay processes after the initial photoexcitation [23,24,25]. XRL, however, is mainly targeting soft matter photoresists that are chemically altered by radiation chemistry and subsequently etched. Furthermore, standard XRL uses relatively large spot sizes and nanostructuring is typically achieved by lithography masks [26,27,28,29,30,31,32]. Very few studies have employed the focused beam of a scanning transmission X-ray microscope (STXM) for maskless direct-write patterning of polymer films [33,34,35,36,37]. Since synchrotron-based X-ray microscopes offer continuously tunable excitation photon energy, it has been shown that within a multilayer of several photoresists consecutively stacked at the same spot, each layer can be addressed separately by the resonant photon energy of the respective polymer resulting in 3D patterning by radiation chemistry [33,34]. The approach is limited to materials with significantly different absorption cross-sections at the chosen incident photon energies, as in transmission geometry, all layers are exposed to the beam at different degrees of focusing. However, such experiments open a completely novel perspective for complex direct-write nanofabrication. It should be mentioned that during the late 1980s and early 1990s several groups attempted to exploit broad-band synchrotron light [38,39,40,41] as well as the monochromatic X-ray beam of a photon-induced scanning Auger microscope [42,43] for additive manufacturing of metal deposits from metal organic precursors. However, due to limited instrumental capabilities, only spatially extended thin films with an optimum of some 10 µm resolution could be produced [43]. 

FXBID exploits the photon energy-selectivity of synchrotron-based XRL and extends it by the idea of depositing metal nanostructures from suitable precursor gases [21,22]. The use of a STXM setup for these experiments offers several advantages. STXM is a raster-scanning technique which avoids the necessity of shadow masks. According to comparable results from polymer lithography the theoretical minimum feature size of the deposited metal structures is mainly determined by the spot size of the incident beam [36,37]. Recent developments in X-ray optics have pushed this parameter below 10 nm [44,45]. Finally, STXM can be employed for an in-situ analysis of the metallic deposits directly after fabrication by means of resonant imaging and near-edge X-ray absorption fine structure (NEXAFS) spectroscopy to evaluate confinement, growth rates, oxidation state and chemical purity [21,22,46,47]. X-ray magnetic circular dichroism (XMCD) can be used to characterize magnetic deposits with respect to their magnetization and coercivity [48]. 

This review presents some basic principles of X-ray optics and X-ray microscopy as well as X-ray beam dosimetry that are relevant for FXBID experiments. In accordance, limitations, challenges and opportunities of additive nanofabrication with FXBID will be discussed with respect to the currently used STXM-based set-up, potential experimental improvements and fundamental limits. Potential applications of FXBID nanofabrication, with a special focus on porous substrates, are discussed. 

## 2. Things to Know about STXM and X-ray Induced Radiation Damage

Following the development of suitable focusing optics for soft X-rays [49,50,51], the first soft X-ray microscopes were installed in the mid-1980s [52,53]. Since then, STXM has developed into a versatile method for characterization of suitably thin specimens from various scientific disciplines, such as biology, medicine, catalysis, material science, magnetism, geology, cosmology, and cultural heritage [54,55,56,57,58,59,60,61,62,63,64]. 

**Figure 1:**
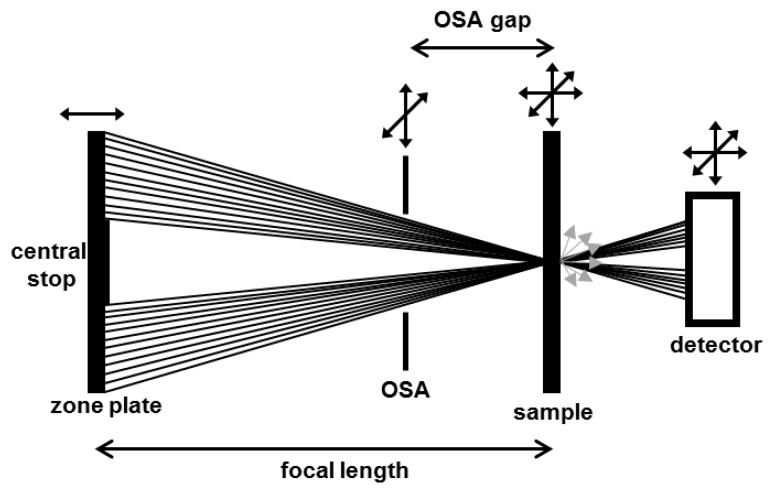
Scheme of main scanning transmission X-ray microscope (STXM) components and their typical degrees of freedom. The monochromatic X-ray beam is focused by a Fresnel zone plate. A proper alignment of central stop and order sorting aperture (OSA) filters undesired diffraction orders. The specimen is raster-scanned through the focal spot of the Fresnel zone plate. Signals are detected in transmission either in form of directly transmitted photons (black) or as undirected secondary radiation (gray arrows).

Figure 1 depicts a scheme of a modern STXM with all basic elements and their respective degrees of freedom [59,65,66]. The monochromatic X-ray beam from the synchrotron source (bending magnet or undulator) is focused by a Fresnel zone plate into a small focal spot. The incident photon energy can be tuned by adjustment of a monochromator grating in front of the microscopy setup. The innermost part the zone plate is not transmitting any light and is referred to as central stop. A proper alignment of central stop and order sorting aperture (OSA) filters undesired diffraction orders from the zone plate optics. Therefore, the OSA has to be smaller than the central stop to block non-diffracted zero order light (progressing parallel to the optical axis) and it has to be placed at an axial position far enough downstream to avoid shadowing of the first order illumination cone, but sufficiently upstream to block higher order light that has a smaller focal length and is already divergent. Presence of unintended diffraction orders with deviating focal lengths leads to aberration artefacts and unnecessary additional radiation dose. The sample is raster-scanned through the focal spot by piezo-scanners and with interferometric position control. Various detectors can be used in STXM. Usually a scintillator assisted photomultiplier tube (PMT) or a photodiode will count transmitted photons. However, also diffraction patterns [67,68,69] or undirected secondary radiation, such as secondary electrons [70,71,72] or fluorescence [73,74,75] can be recorded. STXMs are operated inside an (ultra)-high vacuum chamber to reduce unnecessary photon absorption within the atmosphere.

The heart of an X-ray microscope is the focusing optics. Extended zone plate math is provided in a comprehensible form by [76]. The most important zone plate characteristics in terms of FXBID are the minimum spot size or resolution Δx (coherent illumination assumed), focal length *f,* and depth of focus (*DOF*):

(1) $$\Delta x=\frac{1.22\;\Delta r_{N}}{m}$$

(2) $$f=\frac{4N{\left(\Delta r_{N}\right)}^{2}}{\lambda \;\times \;m}$$

(3) $$DOF=\pm \frac{2{\left(\Delta r_{N}\right)}^{2}}{\lambda \times m^{2}}.$$

*N* is the total number of zones and *m* is the diffraction order (usually only the first order is employed, while higher orders are blocked by the OSA). Equation (1) shows that the spot size in STXM is mainly dependent on the outermost zone width of the applied Fresnel zone plate $\Delta r_{N}$. Since the zones also need to be placed precisely with high aspect ratios of the structures, resolution in X-ray microscopy is mainly an issue of nanofabrication [44,45]. It is very important that *f* is determined by the wavelength of the incident photons in nanometers *λ* and the square of the outermost zone width $\Delta r_{N}$ (Equation (2)). Higher resolutions as well as smaller photon energies both lead to shorter distances of the optics and the sample. Thus, ultra-high resolution is so far limited to photon energies above 600 eV [45,77]. The focal length is also a limiting factor for the use of higher diffraction orders (*m* > 1) for improved spatial resolution. In terms of FXBID experiments the focal length becomes important with respect to the necessity to keep the Fresnel zone plate of the microscope and the precursor gas apart. Otherwise the zone plate would be quickly covered with metal deposits. Ultra-high resolution or very small photon energies compete with the manufacturing of a gas cell sealing around the substrate [21]. The depth of focus is also directly proportional to $\Delta r_{N}$ and indirectly proportional to *λ* (Equation (3)). This has to be taken into account for experiments requiring a certain axial resolution. In FXBID processes DOF might become important when free-standing 3D structures are fabricated or when 3D substrates are functionalized. 

**Figure 2:**
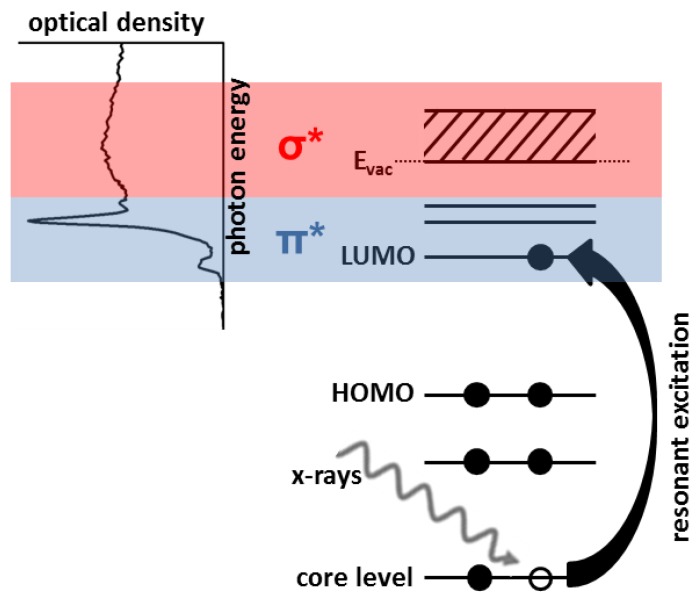
Schematic depiction of electron excitation induced by X-ray absorption and the generation of near-edge X-ray absorption fine structure (NEXAFS) spectra. Resonant soft X-ray illumination induces excitation of core level electrons into unoccupied states. The resulting absorption spectrum exhibits discrete resonant peaks that are a probe of the density and energy levels of respective unoccupied states and, thus, of the chemical state of the excited atom.

STXM can be operated with various contrast mechanisms, but contrast based on material dependent variations in NEXAFS is most common [55,56,59]. Figure 2 presents a scheme of the NEXAFS process. For simplicity, the six electron system carbon is used instead of transition metals with more electron levels. Soft X-ray absorption leads to excitation of a core level electron into an unoccupied state. When the photon energy fits to the energetic distances of the respective energy levels, this excitation is resonant and very likely, resulting in a high absorption coefficient [55,78]. Lowest unoccupied molecular orbital (LUMO) levels are typically well-defined and yield sharp absorption peaks at the low-energy end of the NEXAFS spectrum, while energetically higher levels result in broad peaks. The exact position of the unoccupied states is dependent on the chemical state of the excited atom and, therefore, fine tuning of the excitation energy can yield a strong absorption contrast for slightly chemically different materials [55,59,72]. 

An important limitation in X-ray microscopy is radiation damage. Photo-absorption leads to excitation of electrons and subsequent decay channels that are dominated by the release of low-energy secondary electrons and, thus, the basic processes of radiation damage are very similar to those in electron beam methods [17,24,79]. Several studies have investigated radiation damage in X-ray microscopy with respect to resonant and non-resonant excitation, radiation chemistry and fundamental decomposition principles—mainly for soft organic matter [79,80,81,82,83,84,85,86,87,88,89,90,91,92]. All investigations found that X-ray induced chemistry can be quantitatively correlated with the absorbed dose *d* that is calculated by [83,87]:

(4) $$d=\frac{F\times t\times E}{\varepsilon \times M}$$

(5) $$F=I_{0}\times \left[1-e^{-OD}\right].$$

Within Equation (4) *F* is the number of absorbed photons per second that is calculated from the number of incident photons *I_0_* and the energy-dependent optical density (OD) of the material (Equation (5)). *t* is the acquisition time per area, *E* the photon energy in eV, *ε* is the dimensionless detector efficiency, and *M* is the mass of excited material, typically calculated from excited area and material density. *ε* has to be checked regularly, since it is strongly energy dependent and may vary over time [21,93]. Note, that this consideration of radiation damage with respect to the absorbed dose is different from the point of view in additive lithography that usually relates the amount of deposited material to the number of required photons [21,22]. 

In most STXM studies, X-ray induced radiation chemistry is considered to be avoided and, therefore, referred to as “radiation damage”. The afore-mentioned dosimetric studies usually aim to define critical doses or at least an estimate of experimental limits that should be respected to obtain reliable microscopic and/or spectroscopic data from the investigated specimen. However, under certain circumstances, radiation induced chemical alteration of materials during X-ray microscopic investigation can be exploited for benefits, such as chemically selective direct-write X-ray lithography with polymer resists [33,34,35,36,37], material selective contrast enhancement for improved microscopic imaging of otherwise intricate specimens [90], or using X-rays as release triggers for polymer based microcontainers with potential applications in drug transport, medical imaging and catalysis [94]. FXBID represents another useful application of X-ray induced radiation chemistry. 

## 3. Current FXBID Set-Up

**Figure 3:**
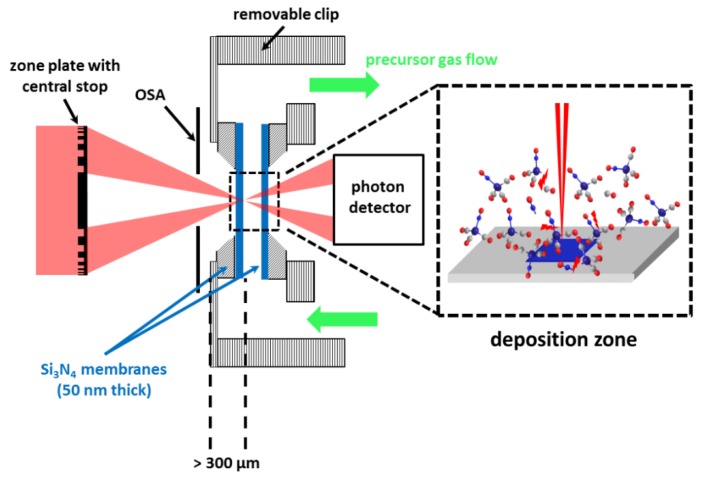
Scheme of the current focused soft X-ray beam induced deposition (FXBID) setup implemented at the PolLux-STXM. The precursor gas is provided within a gas flow cell mainly consisting of two sealed Si_3_N_4_-membranes. The incident X-ray beam is focused onto one of the two membranes for spatially confined deposition of metallic nanostructures.

A scheme of FXBID in its present state of development is depicted in Figure 3. The setup has been implemented into the PolLux-STXM at the Swiss Light Source (SLS) [66]. The precursor gas is provided within an environmental gas flow cell that has been initially designed for in-situ studies in the fields of atmosphere chemistry and catalysis [95,96]. The precursor gas flows between two sealed Si_3_N_4_-membranes with a thickness of 50 nm each. Such membranes are standard sample supports in STXM. After focusing on one of the two membranes, the substrate area selected for deposition is raster-scanned through the focal spot of the Fresnel zone plate by piezo scanners. The transmitted photons are detected down-stream by a scintillator assisted PMT or an avalanche photodiode (APD) [21]. 

The PolLux endstation is usually operated under high vacuum conditions (10^−6^ mbar regime) [66]. The precursor gas is supplied towards the gas flow cell in 3 mm thick tubes. Also, the gas cell itself has to be kept as thin as possible to fit in-between OSA and photon detector. The support plate of the front membrane has a thickness of 100 µm and the Si frame carrying the membrane is 200 µm thick [95]. The membranes should be mounted such that the flat side of the frame is pointing towards the inside of the gas cell. Otherwise the membranes are more prone to breaking when the pressure inside the cell excels the surrounding pressure within the STXM chamber. The membrane frames are glued onto the support with adhesives suitable for vacuum. Therefore, the minimum distance of the deposition zone from the most upstream extension of the gas flow cell towards the optics is currently ~300 µm. 

The OSA has to be placed at a position between the Fresnel zone plate and the focal spot that enables blocking of the already divergent higher diffraction order light (Equation (2)), but avoids shadowing of the illumination cone of the first diffraction order [76]. Therefore, all FXBID experiments so far have been performed with Fresnel zone plates with $\Delta r_{N}$ > 25 nm and still the carbon *K*-edge (280‒320 eV) could not be explored [21,22]. Higher resolving zone plates or photon energies significantly smaller than ~400 eV would lead to a collision of OSA and gas cell at proper focus position. It should also be mentioned that the present setup does not allow for proper measurements of the pressure within the gas cell. The pressure cell is placed outside the PolLux chamber. Due to the small required diameters of the gas cell itself and the supply tubes, a relatively poor pumping cross-section is expected. 

## 4. What We Have Already Learned About FXBID

**Figure 4:**
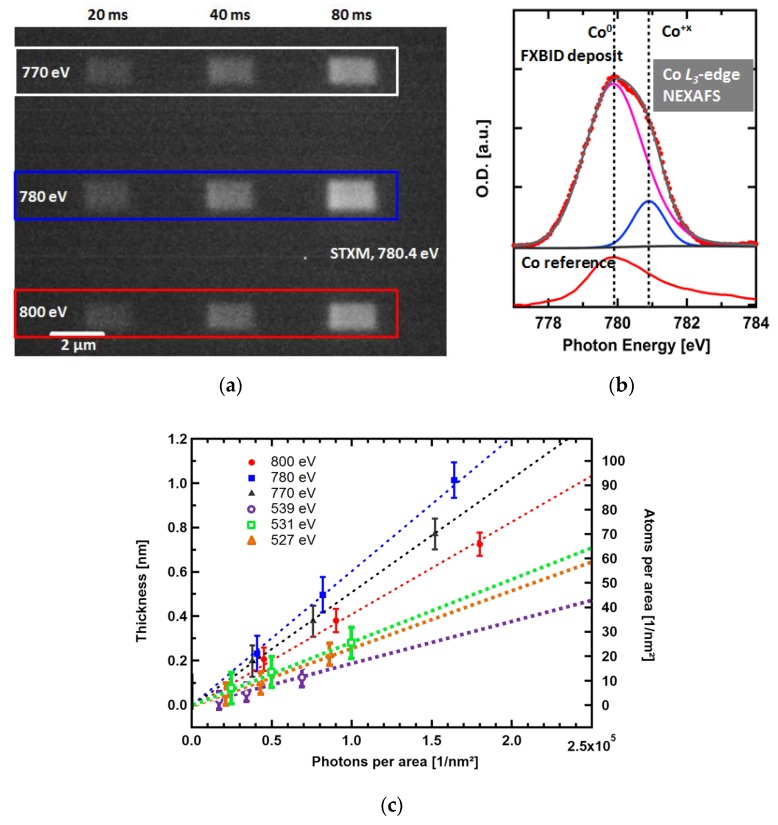
Exemplary data from FXBID with Co(CO)_3_NO and subsequent characterization. (**a**) STXM micrograph (optical density) of FXBID nanostructures deposited with three different incident photon energies around the Co *L*_3_-edge (pre-edge, resonant, and post-edge) and varied illumination time per pixel (100 × 50 pixel per deposit). (**b**) Comparison of Co *L*_3_-edge NEXAFS spectra from an exemplary FXBID deposit with the reference spectrum from a clean Co film. Pink and blue curves represent two individual chemical states and their relative intensities required for fitting of the deposit spectrum. (**c**) Growth rates of FXBID deposits at various photon energies (normalized to energy dependent variations of the incident photon flux). (**a**,**b**) reproduced in revised form from [21] with permission by Royal Society of Chemistry. (**c**) Reprinted from [47] with permission by Cambridge University Press.

FXBID experiments have been successfully performed with cobalt tricarbonyl nitrosyl (Co(CO)_3_NO) [21,22], methylcyclopentadienyl manganese tricarbonyl (MeCpMn(CO)_3_) [22], and meanwhile also with iron pentacarbonyl (Fe(CO)_5_). FXBID yields spatially defined metallic nanostructures that can be in-situ characterized by STXM and NEXAFS in terms of shape, growth rate and chemical purity (Figure 4). There is no evidence of significant proximity effects. However, proximity effects for FEBIP on thin Si_3_N_4_-membranes are also small for low excitation doses due to reduction of the interaction volume [97,98]. The amount of deposited material exhibits direct linear dependence with the dwell time and the supplied precursor pressure. The growth rates vary with incident photon energy. It is concluded that the growth rate at different photon energies is influenced by the absorption cross-section of the respective precursor molecule. 

Photon energy dependent growth rates have been investigated in detail at the following absorption edges:

- Co(CO)_3_NO: Co *L*_3_-edge (~780 eV), O *K*-edge (~530 eV), N *K*-edge (~395 eV),
- Fe(CO)_5_: Fe *L*_3_-edge (~710 eV), O *K*-edge (~530 eV), and
- MeCpMn(CO)_3_: Mn *L*_3_-edge (~640 eV).

At all absorption edges deposits have been fabricated with incident photon energies below, above and on resonance. Since gas-phase spectra of the precursors could not be recorded at the PolLux STXM, the resonant photon energies had to be estimated based on NEXAFS spectra of previously deposited test structures. This is, however, not necessarily correct, since the respective atoms within the deposits might have significantly different chemical states than within the precursor complexes. Nevertheless, in every case significant enhancement of growth rates up to 40% is detected at resonant incident photon energies [21,22] (cf. also Section 5). 

An overview of FXBID studies on Co(CO)_3_NO is presented in Figure 4. Figure 4a shows an in-situ STXM image of FXBID deposits fabricated with various settings in terms of incident photon energy and dwell time per pixel. After recording of Co *L*_3_-edge NEXAFS spectra from test deposits, the photon energies had been chosen as follows:

- 770 eV: Well below Co *L*_3_-edge → no resonant excitation at the Co center of the precursor,
- 780 eV: Close to absorption maximum → resonant absorption at Co center, and
- 800 eV: Well above Co *L*_3_-edge → declining absorption cross-section, mainly photoelectrons.

The STXM micrograph in Figure 4a has been normalized on incident flux to depict local optical density. Therefore, the brighter an area appears in this image, the more Co has been deposited at the respective position. The patterning time is mainly determined by the pixel density and the dwell time per pixel, since the motion of the piezo-scanner is negligible for dwell times >20 ms). It is clearly visible that an extension of the dwell time yields more Co, while resonant excitation at 780 eV excels non-resonant excitation below and above the investigated absorption edge. Co *L*_3_-edge NEXAFS spectra (Figure 4b) of these deposits confirm the presence of Co. Proper fitting of these spectra requires two peaks representing two individual chemical states of Co within the deposits. Comparison with a reference from a clean Co film (prepared by physical vapor deposition) shows that the dominant contribution to the deposit spectrum stems from Co^0^, while a minor portion is Co in undefined oxidized form (Co^+x^).

**Figure 5:**
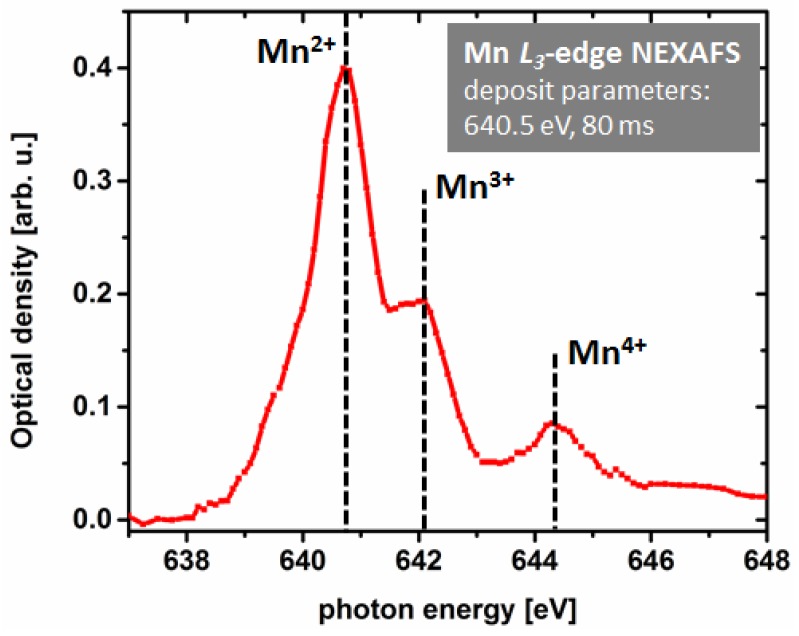
Mn *L*_3_-edge NEXAFS spectrum of an exemplary FXBID deposit from MeCpMn(CO)_3_ (on-resonance deposition). According to reference data at least three individual oxidation states from Mn^2+^ to Mn^4+^ are detected [22].

An inspection of solely Co *L*_3_-edge NEXAFS spectra would suggest a decent chemical purity of the investigated FXBID deposits from Co(CO)_3_NO. However, respective C and O *K*-edge spectra show a high degree of contamination [21]. Especially C *K*-edge NEXAFS is a good indicator to monitor the purity of FXBID nanostructures. It should be mentioned, however, that due to the smaller focal length at the C *K*-edge (~285 eV), the respective substrate has to be removed from the present gas cell set-up. Therefore, C NEXAFS is not recorded in-situ. The relatively high carbon content is attributed to a comparably low base pressure within the gas cell resulting in deposition from residual gas and the CO ligands of the precursor molecule. The detected C *K*-edge NEXAFS spectra are in accordance with spectra from “dead-end”-products of long-time illuminated carbon containing material that has reached a state that cannot be further altered solely by X-ray illumination [89,99]. Due to the relatively high contamination level it was not surprising that XMCD imaging of the respective deposit did not yield any contrast indicating magnetic properties from pure Co or respective pure oxides. The spectroscopic analyses of FXBID deposits from Fe(CO)_5_ and MeCpMn(CO)_3_ yielded similar results in terms of contamination levels. A major difference regarding MeCpMn(CO)_3_ is the lack of Mn^0^ in Mn *L*_3_-edge NEXAFS [22]. Instead, a mixture of various oxidation states from Mn^2+^ to Mn^4+^ was detected (Figure 5). Thus, the chemical analysis suggests that a significant improvement of deposit purity is crucial within subsequent steps of the FXBID project.

Figure 4c provides a comparison of FXBID growth rates from Co(CO)_3_NO at various incident photon energies. Not only resonant and non-resonant excitation are compared, but also excitation around the Co *L*_3_-edge with respective photon energies below, above and on the main resonance of O *K*-edge spectra of previous test deposits. Excitation energies at the N *K*-edge have also been tested [22]. However, the resulting deposition rates were very low. This is attributed to the use of Si_3_N_4_ membranes that are strongly absorbing at the N *K*-edge. Thus, the incident photon flux within the gas cell is significantly reduced. A more detailed evaluation of this issue is intended by using comparable SiO_2_ membranes in future experiments. Deposition at the C *K*-edge was not possible with the current set-up due to spatial constraints from decreasing focal length (Equation (2)). 

For all applied photon energies we detect a linear dependence of deposition with illumination time. After normalization with respect to energy dependent variations of the incident photon flux (including detector efficiency), we detect that resonant excitation always leads to increased deposition rates compared to non-resonant photon energies. The same trend is found for MeCpMn(CO)_3_ at the Mn *L*_3_-edge [22]. When the two depicted absorption edges are compared, we observe that excitation at the Co *L*_3_-edge is more likely to result in deposition compared to the O *K*-edge. A more detailed analysis of this aspect has to take also the different absorption cross-sections at these edges and the higher density of excitable oxygen atoms per volume (the precursor contains 4× more oxygen than cobalt atoms) into account [22]. The sum of both factors should favor fragmentation at the O *K*-edge. Therefore, the experimental results suggest an increased probability of precursor splitting when the initial X-ray induced excitation is localized at the metal center of the precursor complex. This hints on variations of the fragmentation process with excitation photon energy, which might be relevant in terms of potential optimizations of the FXBID process. At the present state more detailed insights into the fundamental processes during excitation at various relevant photon energies are required for a more conclusive interpretation of these observations.

**Figure 6:**
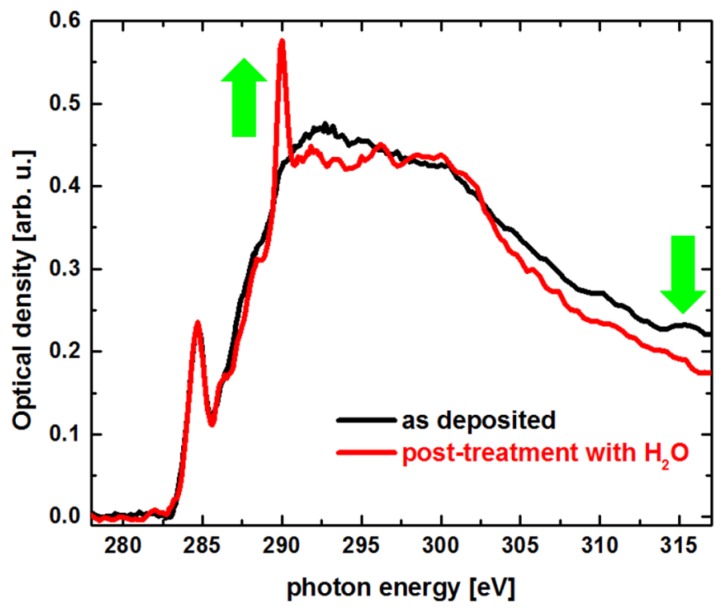
C *K*-edge NEXAFS spectra from Co FXBID deposits (Co(CO)_3_NO precursor) before (black) and after dosing and photo-induced reaction with H_2_O (red). Green arrows highlight the prominent changes in the spectrum. While a post-edge decrease of optical density indicates an overall removal of carbonaceous material, the rise of a peak at ~290 eV is a sign of oxidation.

Subsequent experiments focused on the application of in-situ cleaning procedures. Within a first test, several Co deposits were fabricated under identical FXBID conditions (1 × 0.5 µm, 100 × 50 pixel, 3.0 × 10^−5^ mbar Co(CO)_3_NO, 100 ms dwell time per pixel) and subsequently exposed to 1.0 × 10^−4^ mbar H_2_O. While H_2_O was constantly provided, some deposits were illuminated again with identical pixel density and dwell time as during the initial fabrication cycle. The excitation photon energy for both cycles was 781 eV (Co *L*_3_-resonance). Afterwards NEXAFS spectra were recorded for deposits that were illuminated during H_2_O dosing and for those that were not illuminated. H_2_O exposure without X-ray illumination shows no spectroscopic effect with respect to deposits that were not treated with H_2_O at all. Deposits that were exposed to H_2_O and X-rays simultaneously, however, exhibit significant differences in their respective NEXAFS spectra. Exemplary C *K*-edge NEXAFS spectra are shown in Figure 6. The post-treatment leads to a drop of post-edge optical density. This is a clear indicator for an overall loss of carbonaceous material during the process. Furthermore, a new sharp resonant at ~290 eV appears within the spectrum. Those peaks are a typical fingerprint for the presence of C=O bonds and excitations into π*_C=O_-orbitals [55]. We can conclude that the applied post-treatment procedure induces photo-oxidation of the carbonaceous material within the deposits that is also partially degassing and pumped off. It is not surprising, that the Co NEXAFS spectra, however, also show a significant oxidation of the metallic portion of the respective deposits. Within a subsequent step it must be evaluated whether H_2_O processing can be used to remove carbonaceous material more or less completely. Other reactive gases such as H_2_ might be investigated aiming on not only clean deposit, but also a higher portion of Co^0^ in the final product. 

It was also investigated whether FXBID deposits show significant autocatalytic growth. Under the present experimental conditions this effect was very small, more or less negligible. It took several hours of subsequent precursor dosing to observe a noticeable increase of optical densities of previously deposited FXBID structures. On the one hand this is advantageous at the current status of the project, since deposits do not alter significantly during fabrication of further structures and several deposits can be analyzed in parallel after removal of the precursor gas. However, autocatalytic growth is an important mechanism towards clean deposits in FEBIP [1,15,100,101]. We propose that the current gas flow cell does not provide sufficiently clean conditions for proper autocatalytic growth and any catalytic effect is quenched by rapid contamination of deposited metal atoms.

## 5. Required Next Steps 

It has been confirmed by previous experiments that deposition rates in FXBID are influenced by the incident photon energy [21,22,47]. However, this effect has to be studied in more detail. Transition metal *L*-edge spectra exhibit usually very sharp and intense LUMO peaks excelling non-resonant absorption cross-section by several factors [46,102,103]. The X-ray absorption resonances of the applied precursor molecules should, therefore, be much more dominant compared to the present results of growth rate enhancements of up to 40% [21,22]. To address this topic, it is necessary to record calibrated gas-phase NEXAFS spectra of the intended precursor molecules at a suitable instrument [104,105] and to correlate those spectra with time-dependent density functional theory (TD-DFT) calculations for proper evaluation of the recorded spectral features [106,107]. Such calculations will for each relevant excitation photon energy yield details on the localization of the respective final state levels within the precursor molecules. Recording mass spectra of the fragmentation products at the same photon energies might yield correlations between final state localization and precursor splitting. Furthermore, incident photon energy dependent secondary electron spectra will be measured, since changes in the energy distribution of the emitted secondary spectra should influence precursor decomposition drastically. Deviations from the resonance energies detected for gas-phase precursor molecules and the photon energies yielding maximum FXBID deposition rates might give insights into chemical states of the precursor molecules when absorbed onto the respective substrate.

In terms of purity of the FXBID deposits it is of course possible to apply ex-situ post-processing techniques that are known from the FEBIP community [15,19]. However, it is also intended to evaluate further in-situ cleaning procedures such as co-dosing of reactive gases and in-situ annealing. The latter requires the implementation of heatable Si_3_N_4_-membranes into the gas flow cell. This can be addressed by deposition of Pt or Au wires onto the membrane resulting in a microstructured resistivity heater [108]. 

**Figure 7:**
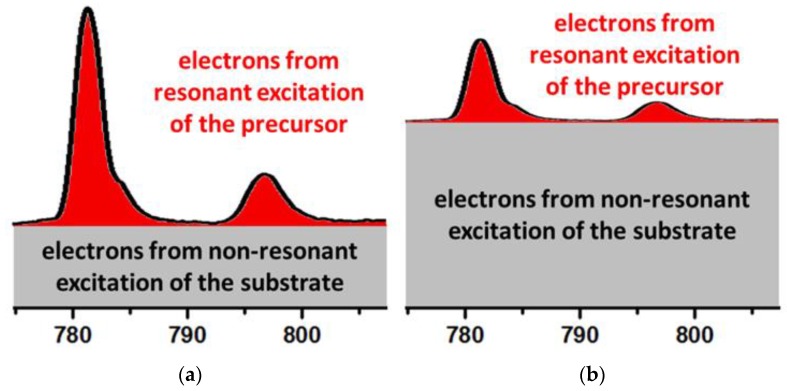
Scheme on the respective contributions of substrate and precursor excitation to overall precursor splitting. (**a**) Excitation of precursor molecules is dominant. FXBID growth rate has a strong dependence on excitation photon energy at the respective absorption edge. (**b**) Excitation of the substrate is dominant. Excitation photon energy has little impact on deposition rate.

The interactions of the precursor and the X-ray beam with the substrate have to be investigated in detail. While these topics have been addressed in depth by the FEBIP community [2,3,15,98,109], there is still very limited literature about the chemical and physical interactions of metal organic precursors with Si_3_N_4_-membranes. Furthermore, it is important to understand to which portions secondary electrons from the substrate are contributing to precursor splitting compared to excitation of the precursor molecules themselves. Figure 7 depicts two potential situations. In Figure 7a decomposition from resonant excitation of the precursor molecules is dominant over the constant background from the substrate. This does not necessarily require that the precursor molecules are emitting more secondary electrons than the substrate. It could also means that the resonantly emitted electrons are in sum more destructive towards the precursor. In this case FXBID would exhibit a strong enhancement of deposition rates for resonant energies and tuning of the excitation energy would yield large effects. In Figure 7b the constant background from the non-resonantly excited substrate is dominant. In that case, photon energy dependent effects would be close to negligible. However, an excitation photon energy close to the N *K*-edge should induce enhanced secondary electron emission from the substrate. The respective yields might differ for various substrates and precursors. Without a major change of the present FXBID setup an evaluation of substrate effect could be performed by using SiO_2_-membranes that have similar technical properties as the standard Si_3_N_4_-membranes. Furthermore, lab-based thermal desorption spectroscopy (TPD) investigations of the intended precursors on such membranes might contribute valuable insights on precursor-substrate interactions by analysis of the respective absorption energies.

In the long term a significant improvement of the chemical purity of the fabricated nanostructures and the successful application of in-situ purification techniques is expected to require a more dedicated setup. The current gas flow cell should be replaced by a two chamber setup with the Fresnel zone plate located in the upstream chamber to be protected from the precursor gas and the substrate in the downstream chamber that can be pumped well into the (U)HV regime. The precursor gas could then be provided by a gas nozzle close to the substrate to achieve a configuration more similar to common FEBIP setups. The two chambers would be connected by a small pinhole along the optical axis that might act as OSA and could even be sealed by a sufficiently transparent membrane window without excessive loss of photon flux. The most sophisticated issue in the design of a two chamber setup is the design of a separator with confined thickness in the area close to the optical axis to allow for a proper alignment of all optical elements and proper focusing of the substrate. In the best case, OSA-to-sample distances <200 µm should be aimed for to enable the use of higher resolving Fresnel zone plates and deposition at the C *K*-edge. Previous experiments on XRL with STXM have shown that the achievable patterning resolution for that approach is in the range of the minimum spot size of the applied Fresnel zone plate [36,37]. In FXBID the mean free path of the occurring secondary electrons is negligible and it is proposed that proximity effects play a minor role. Therefore, the spot size should again be the major limitation for spatial resolution. With the suggested setup improvements, implementation of Fresnel zone plates with 15 nm spot size should be realistic.

An important step towards complex FXBID nanostructures would be the implementation of 3D deposition. STXM is in general a technique for 2D imaging of thin specimens and the respective instruments are designed for such experiments. Only few real 3D studies on suitable objects have been performed employing either focal stack reconstruction [110,111] or tomography [112,113,114]—each dealing with various experimental constraints. However, recent developments are using the laminography concept that is similar to tomography, but uses a rotation angle that is not perpendicular to the optical axis and extend this approach towards soft X-ray 3D imaging [115,116,117]. This method requires a proper 3D scanning of the specimen with retained focus. The hardware activation, controls scripts and reconstruction resources required for laminography imaging can be exploited for controlled and automatized 3D FXBID deposition as well as the subsequent 3D spectroscopic imaging of the resulting nanostructures. 

## 6. Perspectives or: What FXBID Might Be Good For

The major disadvantage of current FXBID is the necessity of a synchrotron source and a STXM setup which heavily restricts availability and accessibility of the technique. However, on the long term it might be possible to transfer the technique to lab-based X-ray sources and simplified instrumentation. If further investigations reveal a limited effect of incident photon energy on the respective deposition rates, it is not necessary to provide a tunable X-ray source for specified technical applications. 

One of the advantages of FXBID is that by tuning of the incident photon energy, the approach provides an adjustable trigger for different precursor splitting rates. Depending on the dimension of this effect, monochromatic focused soft X-rays could be employed for various applications similar to FEBIP, such as 3D nanofabrication, spatially confined substrate functionalization or pattern repair. A potential strength, however, may be photon energy dependent fragmentation of precursor molecules, which has potential to further enhance the in-situ purity of the resulting FXBID deposits. It has been shown that X-ray induced fragmentation of certain relevant precursor molecules exhibits photon energy dependency not only in terms of fragmentation rates, but also fragmentation chemistry, i.e., changing relative intensities of various fragments [103,118,119]. Such effects could be exploited to select photon energies for FXBID that result in a high ratio of low mass fragments (in ideal cases only the metal center itself) versus high mass fragments leading to implantation of a large amount of alien atoms from the ligands into the resulting deposits. 

**Figure 8:**
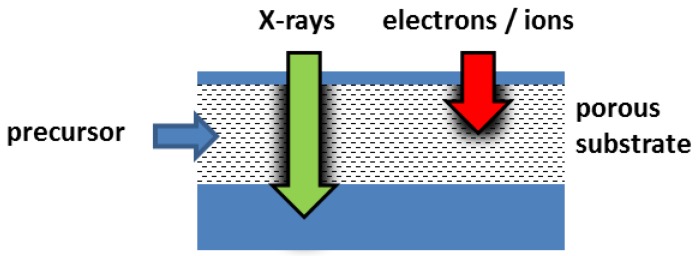
Comparison of penetration depths of X-rays and electrons/ions. In terms of functionalization of porous substrates FXBID allows for depositions zones several µm below the surface of the substrate and might outperform FEBIP/FIBIP.

An inherent advantage of FXBID over FEBIP/FIBIP is illustrated in Figure 8. Focused X-rays have a significantly higher penetration depth than electrons or ions [78]. Depending on the material density and composition, soft X-rays can penetrate matter up to several µm prior to significant decay. Therefore, FXBID could be useful for the functionalization of porous substrates or buried structures with the only limitation that the precursor gas has to reach the intended deposition zone. Deposition in the depth of (nano)porous substrates is a common task in recent atomic layer deposition (ALD) studies targeting on highly reactive catalysts or absorbing agents with extra-large surfaces [120,121,122,123,124,125,126]. FXBID could also be used in terms of a photon assisted ALD process and contribute to increased functionalization rates, deposition from otherwise too stable precursors and spatially confined functionalization. 

Taking all aspects into account FXBID has the potential to provide completely new routes for controlled bottom-up fabrication of complex metallic nanostructures and for the functionalization of sufficiently thin 3D substrates targeting a broad field of applications.
